# Pain in older adults with dementia: Brazilian validation of Pain Intensity Measure for Persons with Dementia (PIMD)

**DOI:** 10.1055/s-0043-1771174

**Published:** 2023-08-30

**Authors:** Mariana Foraciepe, Ana Elisa V. F. Silva, Thais G. Fares, Fânia Cristina Santos

**Affiliations:** 1 Universidade Federal de São Paulo, Disciplina de Geriatria e Gerontologia, Serviço de Dor e Doenças Osteoarticulares, São Paulo SP, Brazil.

**Keywords:** Aged, Dementia, Pain, Pain Assessment, Cognitive Impairment, Pain Measurement, Idoso, Demência, Dor, Avaliação da Dor, Comprometimento Cognitivo, Medição da Dor

## Abstract

**Background** Although there are several ways to assess pain in dementia, there is still a need for tools with better items to assess the presence of pain intensity in these individuals.

**Objective** To validate to Brazilian version of the “Pain Intensity Measure for Persons with Dementia – PIMD-p.

**Methods** Older adults, all demented with impaired verbal communication and exposed to potentially painful situations, were selected from an outpatient clinic and long-term care facility (LTCF). The PIMD-p was applied independently by 2 researchers (E1 and E2) on the same day. Within 14 days, the instrument was reapplied by one of the 2 researchers (E3). The pain intensity reported by participants' caregivers and LTCF nurses were recorded on a verbal numeric pain scale. For the statistical analysis, Cronbach's Alpha, Spearman's Coefficient and intraclass correlation Index were calculated.

**Results** A total of 50 older individuals were selected (mean age 86 years), majority with musculoskeletal pain. The PIMD-p demonstrated good internal consistency according to Cronbach's α (0.838), excellent intra and interobserver reproducibility (0.927 and 0.970, respectively; *p* < 0.001), and convergent validity (strong significant correlations between reported pain intensities and pain indicators on the PIMD-p (except for expressive eyes; corr = 0.106 and *p* = 0.462). A ROC curve was plotted to determine the best cut-off for the PIMD-P, and a score of 7.5 predicted moderate-to-severe pain, with 77.8% sensitivity and 95.7% specificity ( *p* < 0.001).

**Conclusion** The PIMD-p showed satisfactory psychometric properties for measuring intensity of pain in demented older adults with impaired verbal communication.

## Resumo

**Antecedentes** Embora existam várias formas de estimar a dor na demência ainda há necessidade de ferramentas com melhores itens para avaliação da presença e intensidade da dor nesses indivíduos.

**Objetivo** Analisar as propriedades psicométricas de uma ferramenta de avaliação da dor em idosos dementados, a “ *Pain Intensity Measure for Persons with Dementia Portuguese - PIMD-p”* .

**Métodos** Idosos expostos a situações potencialmente dolorosas, sendo esses dementados e com prejuízo na comunicação verbal, foram selecionados em uma unidade ambulatorial e uma instituição de longa permanência, em São Paulo. A PIMD-p foi aplicada por 2 pesquisadores (E1 e E2), de forma separada, num mesmo dia, e, com intervalo de no máximo 14 dias, essa foi reaplicada por apenas um deles (E3), e ainda, foi obtida a intensidade álgica inferida pelos cuidadores dos idosos participantes. Na análise estatística foram utilizados o Alfa de Cronbach, o Coeficiente de Spearman e o Índice de Correlação Intraclasses.

**Resultados** Selecionada uma amostra de 50 idosos com média de idade de 86 anos, a maioria portadora de demência moderada e de dor de origem musculoesquelética. Apuradas para a PIMD-p uma boa consistência interna, segundo o alfa Cronbach (0,838); excelentes reprodutibilidades intra e interobservador (0,927 e 0,970, respectivamente; *p* < 0,001); e uma validade convergente, essa última obtida com as fortes e significativas correlações entre as intensidades dolorosas inferidas e os indicadores de dor do instrumento em estudo (exceto para o indicador “olhar expressivo”; corr = 0,106 e *p* = 0,462).

**Conclusão** *A* PIMD-p se mostrou ser uma ferramenta com propriedades de medida adequadas para avaliar a presença e intensidade álgicas em idosos com demência e com prejuízo na comunicação verbal.

## INTRODUCTION

Due to the major demographic transition in the form of an aging population, the number of dementia cases is set to rise. 1 Currently, there are an estimated 30 million persons living with dementia worldwide, a figure projected to reach 100 million by 2050. 2

Pain is highly prevalent in the older population, especially among demented persons. It has been estimated that 50% of people with dementia and pain are not correctly diagnosed or treated. 1 Individuals experiencing potentially painful situations can develop other symptoms, such as mood (anxiety and depression) and sleep disorders, aggression, agitation and even psychosis, which negatively Impact quality of life and predispose these individuals to disabilities. 2

Some pain-related behaviors in demented persons can be treated inadequately, e.g., with use of antipsychotics for agitation or mechanical restraints which can have serious adverse effects. 3

Evaluating and measuring pain in older people can often be challenging. Traditional tools designed for this purpose depend on the ability of the individual to self-report pain. For instance, the visual analogue scale (VAS), used to determine the intensity of pain, is problematic in the aging population, where around 33% of older person proved unable to answer the VAS. 4 Thus, new tools for pain assessment have been developed for individuals with impaired verbal communication, in an effort to improve treatment and quality of life. 2

Instruments for assessing pain in older adults who are unable to express this verbally have been translated and validated for use in Brazil, such as the PACSLAC, 5 PAINAD 6 and IADIC. 7 These tools assess body language, facial expressions and vocalizations, but which behaviors suggest the intensity of pain have yet to be clearly defined. In fact, there is no single instrument that serves to assess all pain dimensions in the older population and therefore health professionals use those that best suit their place of work. 8

The meta-instrument PIMD was developed to pool a limited set of best items for assessing the intensity of pain in individuals with dementia and some degree of impairment of expression when experiencing potential pain. The PIMD consists of 7 indicators that best correlate to the presence and intensity of pain comprising 3 for facial expressions (highly sensitive and reliable indicators for predicting pain); 1 for positioning, 1 for muscle stiffness, 1 for sighing, and 1 for verbal complaints. 9 The PIMD is a “meta-instrument,” i.e., a tool to “assess the assessments” of pre-existing behaviors indicating pain in dementia. 3 This kind of instrument is used to aggregate findings from a series of evaluations, it also involves an evaluation of the quality of this series of evaluations and its adherence to established good practice in evaluation.

The PIMD was originally developed and validated in English in North America. No publications related to the translation and cross-cultural adaptation of the PIMD in other languages and countries were found. Recently, the PIMD was translated and cross-culturally adapted (PIMD-p) for use in Brazil and was shown to be a very straight forward and practical instrument for measuring pain in demented older individuals). 10 The PIMD-p can be found in the Supplementary Material ( https://www.arquivosdeneuropsiquiatria.org/wp-content/uploads/2023/07/ANP-2023.0020-Supplementary-Material.docx ).

The evaluation of PIMD's psychometric properties in other languages and cultures can yield more details about this new tool.

## METHODS

A methodological, descriptive analytical study was conducted to validate the PIMD-p instrument. All procedures conformed to the ethical standards of the Research Ethics Committee (permit approval number: 0188/2021).

The participants were selected by convenience sampling, a type of non-probability method collecting data for members of the population who are conveniently available for the study. According to some authors, samples of at least 50, and at most 100, individuals are sufficient to assess the psychometric properties of construct measurement instruments. 11 This study involved older adults aged ≥60 years of both sexes recruited from a geriatrics outpatient clinic and a long-term care facility (LTCF), both situated in Sao Paulo city. The inclusion criteria were: participants with dementia of any cause, diagnosed according to the Diagnostic and Statistical Manual - V (DSM-V), 12 with impaired verbal communication, and currently exposed to potentially painful situations (dislocations, bruises, sprains, infections, inflammation, fractures, operations, etc.). Dementia was diagnosed by experienced geriatricians using the Mini Mental State Examination and functionality in daily life (basic and instrumental activities, respectively according to the Katz and Lawton scales). The Clinical Dementia Rating (CDR) scale to measure the degree of dementia was also obtained by those professionals. Exclusion criteria were patients undergoing dialysis, chemotherapy or radiotherapy treatments. The legal representatives of participants selected signed a Free and Informed Consent Form. Data collected included sociodemographics (age, sex, race); information on degree of dementia measured by CDR scale; and etiologies of potential pain. Also, information on pain intensity reported by participants' caregivers and LTCF nurses was collected using the verbal numeric pain scale (classified as mild, moderate or high).

The PIMD-p was applied independently by two researchers (E1 and E2) on the same day. Within 14 days, the instrument was reapplied by one of the researchers (E3), ensuring no different analgesic interventions had been performed over the period.

All statistical analyses were performed using the Statistical Package for Social Science (SPSS), version 17, Minitab 16 and Microsoft Excel 2010. The test of equality of two proportions was used to characterize the distribution and relative frequency of the qualitative variables.

The present study explored the psychometric properties of the PIMD-P including its reliability and validation. Three measures of reliability were obtained: internal consistency (correlation between items); test-retest reproducibility by the same observer (intra-observer reproducability); and reproducibility by different observers (inter-observer reproducibility). 13 Internal consistency was determined using Cronbach's α coefficient (E1), while reproducibility was based on intraclass correlation coefficient (ICC). Convergent validity of the PIMD-p was established using Spearman's test. Also, a ROC curve was plotted for reported pain intensities and total PIMD scores. A 5% significance level was adopted.

## RESULTS

The sample included 50 older individuals, mean age 86.1 years (range 68–100 years), comprising 60% outpatients and 40% LTCF residents, predominantly female (80%) and white (76%). For dementia rating, most participants had moderate (46%) or advanced dementia (42%) ( *p* = 0.687) ( Table 1 ).

**Table 1:** Sample characteristics

Characteristics		N (%)	p-value
Age	Mean 86.1 years		
	Min-max 68–100 years		
Sex	Female	40 (80)	< 0.001
	Male	10 (20)	
Race	White	38 (76)	
	Black	3 (6)	< 0.001
	Brown	9 (18)	< 0.001
Dementia-CDR	1	6 (12)	< 0.001
	2	23 (46)	
	3	21 (42)	0.687
Potentially painful conditions	Muscular	21 (42)	0.110
	Arthritis	26 (52)	0.689
	Vascular disease	4 (8)	< 0.001
	Neurological disease	10 (20)	< 0.001
	Ostomy	7 (14)	< 0.001
	Pressure ulcer or painful skin condition	6 (12)	< 0.001
	Trauma	4 (8)	< 0.001
	Surgery	1 (2)	< 0.001
	Others	4 (8)	< 0.001
Pain – intensity	Mild	23 (46)	
	Moderate	20 (40)	0.545
	Intense	7 (14)	< 0.001

Abbreviation: CDR, clinical dementia rating.

Regarding pain conditions, osteoarticular (52%) and muscular pain (42%) predominated. Pain intensity reported by caregivers and nurses was mainly mild (46%) ( Table 1 ).

Reliability of the PIMD-p according to internal consistency was good, as measured by Cronbach's α (coeff. 0.838). Reliability for intra and inter-observer reproducibility was high and strong, according to the ICC (correlation coefficients 0.927 and 0.970, respectively; *p* < 0.001) ( Table 2 ).

**Table 2:** PIMD-p reproducibility according to ICC

Pain indicator	Interobserver	Intraobserver	Intraobserver	Intraobserver
	ICC	*p* -value	ICC	*p* -value
Bracing	0.934	<0.001	0.958	<0.001
Rigid/stiff	0.927	<0.001	0.976	<0.001
Sighing	0.885	<0.001	0.957	<0.001
Complaining	0.926	<0.001	0.964	<0.001
Grimacing	0.758	<0.001	0.943	<0.001
Frowning	0.663	<0.001	0.930	<0.001
Expressive eyes	0.857	<0.001	0.951	<0.001
Score PIMD-p	0.927	<0.001	0.970	<0.001

Abbreviations: ICC, intraclass correlation coefficient; PIMD-p, pain intensity measure for persons with dementia.

To analyze the psychometric property of the PIMD-p of convergent validity, pain indicators were correlated with pain intensities reported by patients' caregivers and nurses. Results for Spearman's test revealed a strong significant correlation, except for “expressive eyes” (0.106; *p* = 0.462) ( Table 3 ).

**Table 3:** Validity of PIMD-p according to Spearman correlation

Pain indicator	Pain intensity	Pain intensity
	Correlation (r)	*p* -value
Bracing	0.439	0.001
Rigid/stiff	0.505	<0.001
Sighing	0.355	0.011
Complaining	0.605	<0.001
Grimacing	0.519	<0.001
Frowning	0.413	0.003
Expressive eyes	0.106	0.462
PIMD-p Score	0.726	<0.001

Abbreviation: PIMD-P, pain intensity measure for persons with dementia.

A ROC curve was plotted to determine cut-off scores on the PIMD-p. To this end, reported pain intensities were correlated with total pain intensity scores on the PIMD-p. Scores ≥7.5 (0–21) denoted moderate/intense pain intensity, with a sensitivity of 77.8% and specificity of 95.7% (area under curve 0.931; *p* < 0.001) ( Table 4 ). In this study, almost half of the sample had moderate/severe pain (44%).

**Table 4:** Sensitivity and specificity of PIMD-p for pain intensity on ROC curve

Total score PIMD-p	Sensitivity (%)	Specificity (%)
0.5	100	4.3
1.5	100	21.7
2.5	100	39.1
3.5	96.3	56.5
4.5	96.3	65.2
5.5	88.9	82.6
6.5	81.5	82.6
**7.5**	**77.8**	**95.7**
8.5	59	95.7
10.0	33.3	100
11.5	25.9	100
12.5	18.5	100
14.0	11.1	100
15.5	7.4	100
17.5	3.7	100
20.0	0	100
21.0	0	100

Abbreviations: PIMD-P, pain intensity measure for persons with dementia; ROC, Receiver Operating Characteristic.

## DISCUSSION

Pain assessment in older people with dementia and impaired verbal communication remains a challenge for health professionals, since it is unclear which behaviors are most suggestive of pain, unlike for psychological symptoms such as anxiety, agitation and depression.

The present study is the first to analyze the reliability and validity of the PIMD meta-instrument outside its country of origin. This type of investigation is important because, when new measurement instruments are developed, they should undergo broad assessment of their psychometric properties and be analyzed for different population samples. More recently, a systematic review on pain assessment for individuals with advanced dementia in a care home setting identified 17 different tools used worldwide. These instruments included the PIMD, cited for having good psychometric quality and for involving rigorous multidimensional pain assessment. The authors of the review highlighted the need for more studies and tests of existing tools in larger and more diverse samples to better determine their qualities. 14

The present study sample comprised older people from the community and residents of a LTCF. The mean age of the sample was 86.1 years, indicating older participants. Also, individuals predominantly had moderate dementia (46%) and apparent joint and muscular pain etiologies (52% and 42%, respectively). These potential pain etiologies corroborate the data found by Lichtner et al., revealing a higher prevalence of musculoskeletal and osteoarticular pain in older people with dementia. 15

Analyzing the psychometric properties of the PIMD-p, primarily reliability, results confirmed adequate internal consistency (Cronbach's α 0.838). This data corroborates the findings for the original PIMD (Cronbach's α 0.72), while indicating even greater reliability. 3

For PIMD-P reproducibility, excellent results were observed both for intra and inter-observer analyses (ICC 0.970 and 0.927, respectively, both with *p* < 0.001). This high reproducibility of the PIMD-p suggests its utility in clinical practice. Convergent validity for the PIMD-p proved adequate, where the sum of pain intensities calculated for each indicator correlated with the pain intensities reported by caregivers. Strong significant correlation was confirmed, except for the indicator expressive eyes (r 0.106 and *p* = 0.462), where higher PIMD-p scores correlated with greater pain intensities reported by caregivers. In the absence of a gold standard for comparison, convergent validation relative to reported pain intensity was used.

A cut-off point was determined for the PIMD-p due to the fact that pain intensity is a key factor in the choice of analgesic therapy to be used. A ROC curve determined that scores ≥ 7.5, with a sensitivity of 77.8% and specificity of 95.7%, indicated moderate-severe pain ( *p* < 0.001). It was opted for a cutoff point of 7.5 because it greatly optimized Specificity with little reduction in the Sensitivity, thus obtaining a more specific instrument to detect more intense pain.

Some limitations of the study should be noted such as the small sample size. However, the sample did include many oldest-old (mean age 86 years), a group that is still poorly studied, despite being a fast-growing stratum of the population.The PIMD-p proved to be a reliable and valid tool for assessing the presence and intensity of pain in demented older people with difficulties expressing themselves verbally. Therefore, a meta-instrument for pain measurement is now available in Portuguese that has adequate psychometric properties and is both simple and practical. This tool can help health professionals improve care management in the older population with moderate or severe dementia, a group that often includes individuals who are unable to verbally express their pain.
