# Supplementary material for: Pain in older adults with dementia: Brazilian validation of Pain Intensity Measure for Persons with Dementia (PIMD)
Source: Arq Neuropsiquiatr. 2023 Aug 30;81(8):720–4. doi: 10.1055/s-0043-1771174 (PMC10468240; doi:10.1055/s-0043-1771174)
Supplement: Supplementary file 1 — Supplementary Material [file 10-1055-s-0043-1771174-s230020.pdf]

Supplementary Material

Comportamento com descrição	Intensidade do comportamento				
	Ausente	Leve	Moderada	Intensa	Não Aplicável
1. Posicionamento: aplicar o peso de forma desigual para aliviar a pressão de uma parte do corpo, levando mais peso para outra parte.	0	1	2	3	N/A
2. Corpo ou parte dele rígida ou tensa (incluindo rigidez ou tensão relacionadas a contraturas).	0	1	2	3	
3. Suspirar: expiração exagerada e audível, geralmente acompanhada pela elevação e abaixamento dos ombros.	0	1	2	3	
4. Reclamar: expressar verbalmente a insatisfação. Resmungar.	0	1	2	3	
5. Fazer careta: semblante aflito ou distorcido que implica: 1. Sobrancelhas franzidas e/ou olhos entreabertos ou fechados E 2. Um ou mais dos seguintes: a) Lábios apertados; b) Cantos da boca puxados para trás; c) Nariz franzido; d) Bochechas levantadas.	0	1	2	3	
6. Franzir a testa: enrugamento da testa e/ou sobrancelhas caídas/ juntas; podem também estar presentes: cantos da boca para baixo, outras posições da boca não consistentes com franzimento (sorriso, careta).	0	1	2	3	
7. Olha expressivo: olhos bem abertos ou arregalados; sobrancelhas levantadas; olhos entreabertos ou bem fechados.	0	1	2	3	
Pontuação:					
O total de pontos varia de 0–21. Pontuações de 0–7 sugerem nenhuma/dor leve e pontuações maiores que 7 dor moderada/intensa.					

 © 2023. Academia Brasileira de Neurologia. All rights reserved.
